# Photobiostimulatory Effect of a Single Dose of Low-Level Laser on Orthodontic Tooth Movement and Pain

**DOI:** 10.1155/2021/6690542

**Published:** 2021-05-10

**Authors:** Irfan Qamruddin, Mohammad Khursheed Alam, Verda Mahroof, Mubassar Fida, Mohd Fadhli Khamis, Adam Husein

**Affiliations:** ^1^Sindh Institute of Oral Health Sciences, Jinnah Sindh Medical University, Karachi, Pakistan; ^2^Orthodontic Department, College of Dentistry, Jouf University, Sakaka 72721, Aljouf, Saudi Arabia; ^3^Orthodontic Department, Baqai Medical University, Karachi, Pakistan; ^4^Orthodontics Residency Programme, Section of Dental Surgery, Department of Surgery, Aga Khan University, Karachi, Pakistan; ^5^Oral Biology Unit, School of Dental Sciences, Universiti Sains Malaysia, Health Campus, Kota Bharu, Kelantan, Malaysia; ^6^Prosthodontic Unit, School of Dental Sciences, Universiti Sains Malaysia, Health Campus, Kubang Kerian 16150, Kota Bharu, Kelantan, Malaysia; ^7^Hospital Universiti Sains Malaysia, Kubang Kerian16150, Kota Bharu, Kelantan, Malaysia

## Abstract

**Objective:**

To assess the effect of low-level laser applied at 3 weeks intervals on orthodontic tooth movement (OTM) and pain using conventional brackets (CB).

**Materials and Methods:**

Twenty patients with Angle's class II div 1 (10 males and 10 females; aged 20.25 ± 3.88 years) needing bilateral extractions of maxillary first bicuspids were recruited. Conventional brackets MBT of 0.022 in slot (McLaughlin Bennett Trevisi) prescription braces (Ortho Organizers, Carlsbad, Calif) were bonded. After alignment and levelling phase, cuspid retraction began with nitinol closed coil spring on 19 × 25 stainless steel archwire, wielding 150 gram force. 7.5 J/cm^2^ energy was applied on 10 points (5 buccal and 5 palatal) on the canine roots on the investigational side using gallium-aluminum-arsenic diode laser (940 nm wavelength, iLase™ Biolase, Irvine, USA) in a continuous mode. Target tissues were irradiated once in three weeks for 9 weeks at a stretch (T0, T1, and T2). Patients were given a feedback form based on the numeric rating scale (NRS) to record the pain intensity for a week. Silicon impressions preceded the coil activation at each visit (T0, T1, T2, and T3), and the casts obtained were scanned with the Planmeca CAD/CAM™ (Helsinki, Finland) scanner.

**Results:**

The regimen effectively accelerated (1.55 ± 0.25 mm) tooth movement with a significant reduction in distress on the investigational side as compared to the placebo side (94 ± 0.25 mm) (*p* < 0.05).

**Conclusions:**

This study reveals that the thrice-weekly LLLT application can accelerate OTM and reduce the associated pain.

## 1. Introduction

Fixed orthodontic treatment is a lengthy and time-consuming process and on average takes 12–36 months [1] and is associated with adverse outcomes, particularly pain and difficulty to carry out oral hygiene practices. Prolong treatment and difficulty is to maintain proper oral hygiene on mobile, and tender dentition is not only detrimental to the teeth and surrounding periodontal tissues but also influence patient compliance and productivity of the healthcare professionals [2]; therefore, orthodontic contemporaries are toiling on efficient and fast force delivery mechanics and approaches [3].

Interventions such as a local injection of pharmacological agents, use of magnets or direct current, and invasive surgical approaches (corticotomy) trim the total treatment time by stimulating bone remodelling but at the expense of either increased patient's suffering or systemic side effects [4].

Low-level laser therapy (LLLT) is being used to alleviate musculoskeletal pain for decades. However, its use in dentistry is gaining popularity as a noninvasive and safe modality. Moreover, its anti-inflammatory effects and potential to induce peripheral neural blockage makes it a suitable candidate for postactivation pain and healing of tissues [5].

LLLT, when applied at correct intensity and duration, has been proven to amp up tissue healing by increasing cell proliferation (fibroblasts, osteoclasts, and osteoblasts), angiogenesis, and collagen synthesis [6]. At the molecular level, red or infrared light donates free electrons to the electron transport chain in mitochondria to curb the oxidative stress and generate more ATP [7]. This cascade of reactions, in turn, triggers growth signalling pathways and upregulates various transcription factors [8], with an overall increase production of growth factors [5].

A handful of researchers document the effect of LLLT on OTM, but the diversity of results pertains to different laser specifications, dosages, points of application, and intervals of application results [9–12], therefore requiring further insight into precise and specific emissions of radiation to get optimal results.

This research was aimed at providing a single dose of LLLT application to expedite tooth movement and lessen the discomfort associated with it.

## 2. Materials and Methods

This was the placebo-controlled clinical study, and the research was conducted in the Department of Orthodontics at Baqai Medical University, Pakistan. Twenty-two patients, age ranging from 12 to 30 years (10 males and 10 females), with healthy medical and dental status (no missing or impacted teeth except third molars) and no history of orthodontic treatment were recruited in the trial.

The inclusion criteria were patients with 1/4 or half cusp, molar class II division 1 warranting extraction of upper bicuspids on both sides. Patients who require lower premolar extraction were excluded from the study because simultaneous lower canine retraction interferes with the retraction of upper canines. Patients with TMJ problems or taking medicines that modify bone turnover or interferes with tooth retraction, e.g., NSAIDs, bisphosphonates, and corticosteroids, were disqualified.

Regular diagnostic orthodontic records were collected and thoroughly examined after the approval from the ethical board of Baqai Medical University. The whole procedure was verbally explained, and assent form was signed from the patients and legal guardians of minors.

Split-mouth design was chosen by flipping a coin to circumvent individual bias, randomly assigning one side as an experimental and the other placebo group.

After all the necessary procedures, banding and bonding were carried out. MBT (McLaughlin Bennett Trevisi) of 0.22 inch slot prescription braces (Ortho Organizers, Carlsbad, Calif) were bonded. The first stage of levelling and alignment was commenced with 0.014 inch heat-activated nitinol (NiTi) wire and after that by 0.016 inch NiTi, 0.017 × 0.025 in NiTi, 0.019 × 0.025 inch NiTi, and 0.019 × 0.025 SS as the final working wire. The first bicuspid was then extracted at day 21, and individual canine retraction began with 6 mm close coil NiTi spring, stretched to 150 gm force, measured with the orthodontic dynamometer (Forestadent, Germany) and secured with a ligature tie between the power arm of canine and first molar band.

**Figure 1:**
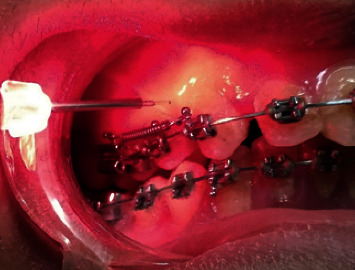
Laser application.

LLLT irradiation was applied soon after the placement of spring on the experimental side and was held at the placebo side without turning it on (Figure 1). The springs were activated at a three-week interval. Silicon impressions were taken before the first activation (T0) and repeated at every appointment before activation for nine consecutive weeks, i.e., T1, T2, and T3. Dental casts were scanned with the Planmeca CAD/CAM lab scanner for further analysis.

### 2.1. Laser Specification

Ga-Al-As diode laser (Ilase, USA) operated at 940 nm wavelength in a continuous, uninterrupted beam of light was used. Irradiations were delivered through the 0.04 cm^2^ diameter optical fibre tip in light contact with the oral mucosa.

The target area was irradiated on ten sites, five points buccally and five palatally, for 3 secs each. The areas were as follows:

- Mesial and distal to the cervical area of the canine
- Mesial and distal to the apical area of the canine
- One point in the middle of the root

The power output set at 100 mW for 3 sec at each point made the cumulative of 7.5 J/cm^2^ energy density. A separate room with loud music was reserved for the procedure. All the personnel wore protective shades near irradiated laser (patient, assistant, and dentist). To avoid the carryover effect, a plastic shield of the same wavelength as that of the laser was used.

### 2.2. Measurements

#### 2.2.1. Rate of Canine Movement

To assess the effectiveness of regimen, the comparison of right and left sides was made, i.e., experimental and placebo at T0, T1, T2, and T3. A system suggested by Gebauer was used, and *x* and *y* marks were drawn on 3D imageries of study cast [13]. *Y*-axis was drawn a parallel to raphe line, and medial end of the prominent rugae marked the plane for the *x*-axis. The distance covered by canine was given by measuring the distance from *x* coordinate to the most distal point on canine on both the sides, and the two reading were later compared for the effectiveness.

### 2.3. Postactivation Pain

The analgesic effect of the LLLT evaluated by a feedback form was designed based on 11 points (from 0 to 10) numeric rating scale (NRS) where zero indicates no discomfort and 10 excruciating, terrible pain.

The form was given to the patient at each appointment and collected at the subsequent show-up. They were instructed to record the pain four hours after the activation and thereupon every 24-hour interval for the next 7 days. Patients were told not to take any analgesics if needed and advised to jot it down.

### 2.4. Statistical Analysis

Data were put in and interpreted on the SPSS 20.0 version. The Mann–Whitney *U* test was performed to compare the canine movement and Kruskal–Wallis test for pain comparison.

## 3. Results

### 3.1. Rate of Canine Retraction

22 patients were recruited in the study, and two of them later were disqualified due to spring dislodgement and use of analgesics.

The Mann–Whitney *U* test shows a statistically significant acceleration in canine movement on the experimental side in comparison to the placebo group (Table 1). In 9 weeks, canine achieved 4.67 mm movement on the experimental side and 2.87 mm on the placebo side. Moreover, the average cuspid displacement in the experimental and placebo groups was 1.55 ± 0.25 mm and 0.94 ± 0.25 mm, respectively. The overall rate of displacement in the experimental group exceeded 1.66 times than the placebo group.

*Pain*. Most patients experienced the highest level of discomfort on the day the spring got activated. Females reported heightened pain sensitivity as compared to males. A significant reduction in pain in the lased group for initial 2 days was found. No difference was noted in the remaining days of the week.

**Figure 2:**
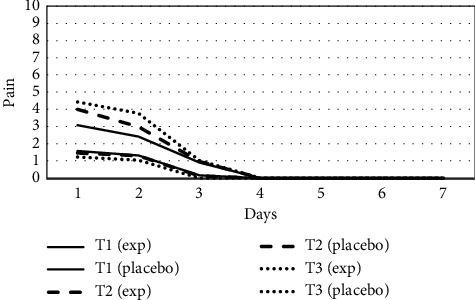
Comparison of pain among experimental side and placebo side in group A at T1, T2, and T3.

Highest pain scores were recorded in the placebo side at T3 (Figure 2). There was a significant reduction in pain on the experimental side at all stages of treatment (T1, T2, and T3) as the level of pain was significantly higher on placebo sides.

## 4. Discussion

This research was undertaken to appraise the effectiveness of a single dose of laser on OTM and twinge using conventional brackets, applied at 3 weeks' interval.

Pain and rate of movement are subjective quantities and are greatly influenced by age, gender, hormones, pain threshold, and anatomic variations [14]. Therefore, the split-mouth design was considered to circumvent chances of error. However, it holds an inherent disadvantage of the carryover effect. For that, a plastic shield of the same wavelength as that of the laser was placed in the midline.

To maximize the effectiveness of placebo design, the whole protocol was carried out in a separate room, and loud music was played on to mingle it with the beeping sound of the laser. None of the patients complained about the heating, burning sensation, or any form of discomfort.

A bunch of researchers has employed single-blind trials with split-mouth design, but none of them brought the carryover effect and blinding into consideration [9, 10, 15–18].

Ga-Al-As semiconductor diode with 940 nm wavelength was used due to its deeper depth of penetration, about its low absorption coefficient in haemoglobin and water and its subsequent ability to stimulate osteoblastic activity on the target tissue [19]. Several previous authors also used Ga-Al-As with the wavelength ranging from 650 nm to 860 nm. Energy output, however, varied in all the studies and led to speckled results [2, 9–11, 15–18, 20].

In this study, energy dose was kept 7.5 J/cm^2^ at each point as low doses impart biostimulatory effects [4, 21].

Research studies catering laser photobiostimulation on OTM reveals that patients had to make some additional visits along with the regular ones for the regimen, making it difficult for them to stick to it [9–12, 22]. In our research, LLLT was applied once in three weeks, and a profound acceleration was observed because LLLT works best to stimulate bone remodelling if applied within 48 hours after force application [23]. This is in agreement with few previous research studies which found a single dose of LLLT to be efficient in accelerating OTM and reducing associated pain [24–26].

Since bone remodelling is directly related to cytokine production, LLLT stimulates bone remodelling by accelerating the production of IL-*β*, and receptor activator which is crucial for osteoclastic activity on day 2 or 3 after laser application [27].

The overall rate of canine movement was 1.65 times greater in the present study. However, Youssef and Sousa concurred with twice the rate and Doshi-Mehta, and Bhad Patil found it to be 1.3 times faster than the control group [10–12]. Others found no significant acceleration [20, 28]. Qamruddin et al. reported 2.02 times acceleration in canine movement; however, more acceleration attributes to the use of frictionless self-ligating brackets in the study [24].

In previous research studies, the measurements were made from canine cusp tip or distal surface of canine to mesiobuccal cusp of the first molar with a digital calliper, held directly on the dental cast [11, 12, 15]. Curved palatal anatomy, rotated molars, and difficulty in holding the calliper directly over the cast pose difficulty in recording the precise measurements, therefore, in this research, we took medial part of most prominent rugae as a stable reference landmark [29] and scanned the respective models through the CAD/CAM scanner [20] to assure the accuracy in measurements.

To assess the pain levels in patients undergoing LLLT therapy, a questionnaire (feedback form) was formulated using NRS in contrast to others who employed a visual analogue scale (VAS). NRS is more accurate and easily understood by patients of any age and educational background [30].

In the present study, the pain rating was very low in the lased as well as the placebo group. Highest pain scores were reported on day 1 of coil activation, which agrees with the previous studies [31–33]. Experimental and placebo groups showed a significant difference in the level of pain. This is well supported by some studies which documented the pain-alleviating effect of LLLT [34–37] experienced during canine retraction [11, 12]. Almallah et al. also revealed a 12% decrease in discomfort after a single dose of low-level helium-neon laser in the experimental group [34]. Few more studies evinced the analgesic effects laser. However, the effects were on pain linked with the insertion of initial archwire [31–33]. Some authors find a nonsignificant difference in pain associated with canine retraction between the control and lased groups [9, 20].

## 5. Conclusion

Application of LLLT at regular orthodontic visits (3 weeks intervals) accelerates OTM and decreases the pain significantly. Hence, the above regimen can be implemented in fixed orthodontic treatment to avoid the risk of patient and operator burnout.

## Figures and Tables

**Table 1:** Median values and standard deviation of canine movements in experimental and placebo groups with confidence interval and *p* values.

	Experimental side (mean (SD))	95% CI		Placebo side (mean (SD))	95% CI		*P* value
		Lower bound	Upper bound		Lower bound	Upper bound	
T0-T1	1.79 (0.25)	1.63	1.95	1.12 (0.21)	0.98	1.25	<0.001^*∗*^
T1-T2	1.59 (0.29)	1.40	1.78	0.91 (0.19)	0.79	1.03	<0.001^*∗*^
T2-T3	1.29 (0.25)	1.12	1.45	0.80 (0.24)	0.64	0.95	<0.001^*∗*^

^*∗*^Significant at *p* < 0.05 (Mann–Whitney *U* test).

## Data Availability

The data used to support the findings of this study are presented within the results section as tables and are available from the corresponding author upon request.

## References

[1] Fink D. F., Smith R. J. (1992). The duration of orthodontic treatment. *American Journal of Orthodontics and Dentofacial Orthopedics*.

[2] Turbill E. A., Richmond S., Wright J. L. (2001). The time-factor in orthodontics: what influences the duration of treatments in national health service practices?. *Community Dentistry and Oral Epidemiology*.

[3] Jawad M. M., Husein A., Alam M. K., Hassan R., Shaari R. (2014). Overview of non-invasive factors (low level laser and low intensity pulsed ultrasound) accelerating tooth movement during orthodontic treatment. *Lasers in Medical Science*.

[4] Qamruddin I., Alam M. K., Khamis M. F., Husein A. (2015). Minimally invasive techniques to accelerate the orthodontic tooth movement: a systematic review of animal studies. *BioMed Research International*.

[5] Karu T. I., Kolyakov S. F. (2005). Exact action spectra for cellular responses relevant to phototherapy. *Photomedicine and Laser Surgery*.

[6] Zahra S. E., Elkasi A. A., Eldin M. S., Vandevska-Radunovic V. (2009). The effect of low level laser therapy (LLLT) on bone remodelling after median diastema closure: a one year and half follow-up study. *Orthodontic Waves*.

[7] Greco M., Guida G., Perlino E., Marra E., Quagliariello E. (1989). Increase in RNA and protein synthesis by mitochondria irradiated with helium-neon laser. *Biochemical and Biophysical Research Communications*.

[8] Chen A. C., Arany P. R., Huang Y.-Y. (2011). Low-level laser therapy activates NF-kB via generation of reactive oxygen species in mouse embryonic fibroblasts. *PLoS One*.

[9] Limpanichkul W., Godfrey K., Srisuk N., Rattanayatikul C. (2006). Effects of low-level laser therapy on the rate of orthodontic tooth movement. *Orthodontics and Craniofacial Research*.

[10] Da Silva Sousa M. V., Scanavini M. A., Sannomiya E. K., Velasco L. G., Angelieri F. (2011). Influence of low-level laser on the speed of orthodontic movement. *Photomedicine and Laser Surgery*.

[11] Doshi-Mehta G., Bhad-Patil W. A. (2012). Efficacy of low-intensity laser therapy in reducing treatment time and orthodontic pain: a clinical investigation. *American Journal of Orthodontics and Dentofacial Orthopedics*.

[12] Youssef M., Ashkar S., Hamade E., Gutknecht N., Lampert F., Mir M. (2008). The effect of low-level laser therapy during orthodontic movement: a preliminary study. *Lasers in Medical Science*.

[13] Häsler R., Schmid G., Ingervall B., Gebauer U. (1997). A clinical comparison of the rate of maxillary canine retraction into healed and recent extraction sites—a pilot study. *The European Journal of Orthodontics*.

[14] Eslamian L., Borzabadi-Farahani A., Edini H. Z., Badiee M. R., Lynch E., Mortazavi A. (2013). The analgesic effect of benzocaine mucoadhesive patches on orthodontic pain caused by elastomeric separators, a preliminary study. *Acta Odontologica Scandinavica*.

[15] Genc G., Kocadereli İ., Tasar F., Kilinc K., El S., Sarkarati B. (2013). Effect of low-level laser therapy (LLLT) on orthodontic tooth movement. *Lasers in Medical Science*.

[16] Cruz D. R., Kohara E. K., Ribeiro M. S., Wetter N. U. (2004). Effects of low-intensity laser therapy on the orthodontic movement velocity of human teeth: a preliminary study. *Lasers in Surgery and Medicine*.

[17] Gui L., Qu H. (2008). Clinical application of low energy laser in acceleration of orthodontic tooth movement. *Journal of Dalian Medical University*.

[18] Cheng-Wei X., Ze-Jun Z., Zhao J. (2006). The effect of low energy laser on accelerating orthodontic tooth movement. *Medical Journal of Qilu*.

[19] Hamblin M. R., Demidova T. N. (2006). Mechanisms of low level light therapy. *Proceedings of SPIE—The International Society for Optical Engineering*.

[20] Heravi F., Moradi A., Ahrari F. (2014). The effect of low level laser therapy on the rate of tooth movement and pain perception during canine retraction. *Oral Health and Dental Management*.

[21] Ge M. K., He W. L., Chen J. (2015). Efficacy of low-level laser therapy for accelerating tooth movement during orthodontic treatment: a systematic review and meta-analysis. *Lasers in Medical Science*.

[22] Jerrold L., Naghavi N. (2011). Evidence-based considerations for determining appointment intervals. *Journal of Clinical Orthodontics: JCO*.

[23] Saito S., Shimizu N. (1997). Stimulatory effects of low-power laser irradiation on bone regeneration in midpalatal suture during expansion in the rat. *American Journal of Orthodontics and Dentofacial Orthopedics*.

[24] Qamruddin I., Alam M. K., Mahroof V., Fida M., Khamis M. F., Husein A. (2017). Effects of low-level laser irradiation on the rate of orthodontic tooth movement and associated pain with self-ligating brackets. *American Journal of Orthodontics and Dentofacial Orthopedics*.

[25] Qamruddin I., Alam M. K., Fida M., Khan A. G. (2016). Effect of a single dose of low-level laser therapy on spontaneous and chewing pain caused by elastomeric separators. *American Journal of Orthodontics and Dentofacial Orthopedics*.

[26] Qamruddin I., Alam M. K., Abdullah H., Kamran M. A., Jawaid N., Mahroof V. (2018). Effects of single-dose, low-level laser therapy on pain associated with the initial stage of fixed orthodontic treatment: a randomized clinical trial. *The Korean Journal of Orthodontics*.

[27] Kaku M., Motokawa M., Tohma Y. (2008). VEGF and M-CSF levels in periodontal tissue during tooth movement. *Biomedical Research*.

[28] Domínguez A., Gómez C., Palma J. C. (2015). Effects of low-level laser therapy on orthodontics: rate of tooth movement, pain, and release of RANKL and OPG in GCF. *Lasers in Medical Science*.

[29] Gebauer U. (1977). Elektronische Mess-und Rechenanlage zur arcogrammetrischen Modelldiagnostik und zum Auswerten von Fernröntgenbildern. *Schweizerische Monatsschrift für Zahnheilkunde*.

[30] Fujiyama K., Deguchi T., Murakami T., Fujii A., Kushima K., Takano-Yamamoto T. (2008). Clinical effect of CO_2_ laser in reducing pain in orthodontics. *The Angle Orthodontist*.

[31] Harazaki M., Isshiki Y. (1997). Soft laser irradiation effects on pain reduction in orthodontic treatment. *The Bulletin of Tokyo Dental College*.

[32] Tortamano A., Lenzi D. C., Haddad A. C. S. S., Bottino M. C., Dominguez G. C., Vigorito J. W. (2009). Low-level laser therapy for pain caused by placement of the first orthodontic archwire: a randomized clinical trial. *American Journal of Orthodontics and Dentofacial Orthopedics*.

[33] Deshpande P., Patil K., Mahima V. G., Shivalinga B. M., Suchetha M., Ranjan A. (2016). Low-level laser therapy for alleviation of pain from fixed orthodontic appliance therapy: a randomized controlled trial. *Journal of Advanced Clinical & Research Insights*.

[34] Almallah M. M. E., Hajeer M. Y., Almahdi W. H., Burhan A. S., Latifeh Y., Madkhaneh S. (2020). Assessment of a single versus double application of low-level laser therapy in pain reduction following orthodontic elastomeric separation: a randomized controlled trial. *Dental and Medical Problems*.

[35] Almallah M. M., Almahdi W. H., Hajeer M. Y. (2016). Evaluation of low level laser therapy on pain perception following orthodontic elastomeric separation: a randomized controlled trial. *Journal of Clinical and Diagnostic Research: JCDR*.

[36] Alam M. K. (2019). Laser-assisted orthodontic tooth movement in Saudi population: a prospective clinical intervention of low-level laser therapy in the 1st week of pain perception in four treatment modalities. *Pain Res Management*.

[37] Qamruddin I., Khan A. G., Asif F. M. (2020). Pain perception and rate of canine retraction through self-ligating brackets and conventional elastomeric ligation system: a split mouth study. *Pesquisa Brasileira em Odontopediatria e Clínica Integrada*.

